# Cellular Energy Allocation to Assess the Impact of Nanomaterials on Soil Invertebrates (Enchytraeids): The Effect of Cu and Ag

**DOI:** 10.3390/ijerph120606858

**Published:** 2015-06-16

**Authors:** Susana I. L. Gomes, Janeck J. Scott-Fordsmand, Mónica J. B. Amorim

**Affiliations:** 1 Department of Biology & CESAM, University of Aveiro, Aveiro 3810-193, Portugal; E-Mail: mjamorim@ua.pt; 2 Department of Bioscience, Aarhus University, Vejlsovej 25, PO BOX 314, Silkeborg DK-8600, Denmark; E-Mail: jsf@bios.au.dk

**Keywords:** energy available, energy consumption, energy budget, Oligochaeta, nanomaterials

## Abstract

The effects of several copper (Cu) and silver (Ag) nanomaterials were assessed using the cellular energy allocation (CEA), a methodology used to evaluate the energetic status and which relates with organisms’ overall condition and response to toxic stress. *Enchytraeus crypticus* (Oligochatea), was exposed to the reproduction effect concentrations EC_20_/_50_ of several Cu and Ag materials (CuNO_3_, Cu-Field, Cu-Nwires and Cu-NPs; AgNO_3_, Ag NM300K, Ag-NPs Non-coated and Ag-NPs PVP-coated) for 7 days (0-3-7d). The parameters measured were the total energy reserves available (protein, carbohydrate and lipid budgets) and the energy consumption (Ec) integrated to obtain the CEA. Results showed that these parameters allowed a clear discrimination between Cu and Ag, but less clearly within each of the various materials. For Cu there was an increase in Ec and protein budget, while for Ag a decrease was observed. The results corroborate known mechanisms, e.g., with Cu causing an increase in metabolic rate whereas Ag induces mitochondrial damage. The various Cu forms seem to activate different mechanisms with size and shape (e.g., Cu-NPs *versus* Cu-Nwires), causing clearly different effects. For Ag, results are in line with a slower oxidation rate of Ag-NMs in comparison with Ag-salt and hence delayed effects.

## 1. Introduction

Several studies have reported negative effects on soil invertebrate population measures, e.g., on survival and reproduction, caused by copper (Cu) and silver (Ag) nanomaterials (NMs). For example, Cu-NPs studies showed negative effects on *Enchytraeus albidus* and *Eisenia fetida* reproduction [1,2]; for Ag, Gomes *et al.* [3] showed that *E. albidus* had decreased reproduction following exposure to polyvinylpyrrolidone (PVP)-coated Ag-NPs.

Such negative impacts are mediated through various mechanisms, e.g., through oxidative stress and subsequent damage, where the reported effect concentrations are lower and the impact is observed at shorter periods of exposure than for effects such as survival and reproduction [4,5,6]. Apart from affecting specific pathways of toxicity, the handling of NMs by the organisms may also impact (and be impacted by) the energy allocation which is otherwise used to maintain a normal body function. The Cellular Energy Allocation (CEA) methodology is an approach that integrates the energy available and energy consumption of an organism, and has been established as a good marker of exposure to stress [7,8,9]. The rationale is that organisms can mobilize their energy reserves to deal with stressful conditions e.g., detoxification processes. However, this has consequences for other biological functions such as growth and/or reproduction [7]. Hence, changes in the CEA may partly show exposure and also be linked to effects at higher levels of biological organization [8,9,10,11,12].

In the present study we aimed to investigate the effects of four Cu and four Ag materials in terms of energy costs/allocation, with the parameters measured being: protein, carbohydrate and lipid contents (which constitute the energy available—Ea) and the energy consumption—Ec in the form of electron transport system (ETS) activity, after three and seven days of exposure. The integrated ratio of Ea and Ec corresponds to the net energy budget of the organisms or CEA. This study was performed using the soil standard organism *E. crypticus* (Enchytraeidae, Oligochaeta) [13,14,15]. To link energy cost/allocation to population level responses all materials were tested at the concentrations which caused no effects (controls), a 20% (EC_20_) and a 50% (EC_50_) on the reproductive output for the organisms.

## 2. Materials and Methods

### 2.1. Test Species

The test species *Enchytraeus crypticus* Westheide and Graefe, 1992, was used. Individuals were cultured in Petri dishes containing agar medium, consisting of a sterilized mixture of four different salt solutions (CaCl_2_·2H_2_O; MgSO_4_; KCl; NaHCO_3_) and a Bacti-Agar medium (Agar No. 1, Oxoid, Lancashire, UK). The cultures were kept under controlled conditions, at 19 ± 1 °C and photoperiod 16:8 h light:dark. Organisms were fed on ground and autoclaved oats twice a week.

### 2.2. Test Soils

For the copper exposure the test soil was collected at the Hygum site (Jutland, Denmark). The general physicochemical characteristics of the Hygum study-site soil are as follows: 20%–32% coarse sand (>200 µm), 20%–25% fine sand (63–200 µm), 11%–20% coarse silt (20–63 µm), 12%–20% silt (20–20 µm), 12%–16% clay (<2 µm), 3.6%–5.5% organic matter, Cation Exchange Capacity 10–13 meq/100 g, pH = 5, N 0.25%–0.31% and P 0.10%–0.12%. The clay mineralogy analysed by X-ray diffraction were dominated by illite, kaolinite, chlorite and vermiculite. For further details see [16].

The Hygum site soil has been exposed to contamination with CuSO_4_ due to activities of timber preservation, which ceased more than 80 years ago. There is a well-known Cu gradient along the field, ranging from natural background levels of 15 up to 2900 mg Cu/kg dry soil, this being an excellent natural study site [16]. Soil was sampled in the field to a depth of 20 cm. To exclude soil fauna, the soil was dried at 80 °C for 24 h in an oven (Memmert, Type UL40, Braunschweig, Germany), and then sieved through a 2 mm mesh to remove larger particles.

For the silver exposure the natural standard soil LUFA 2.2 (LUFA, Speyer, Germany) was used. The main characteristics can be described as follows: pH (0.01 M CaCl_2_, ratio 1:5 w/v) = 5.5, organic matter = 1.77 meq/100g, CEC (cation exchange capacity) = 10.1%, WHC (water holding capacity) = 41.8% grain size distribution of 7.3% clay, 13.8% silt, and 78.9% sand.

### 2.3. Test Chemicals and Characterization

The Cu forms tested included copper nitrate (Cu(NO_3_)_2_·3H_2_O > 99%, Sigma Aldrich, St. Louis, MO, USA) and two Cu NMs, *i.e.*, Cu-nanoparticles (Cu-NPs, 99.8%, 20–30 nm, American Elements, Los Angeles, CA, USA) and Cu-nanowires (Cu-Nwires) which were synthesized in house following the procedure described by [17] by reduction of copper (II) nitrate with hydrazine in alkaline medium.

The Ag forms tested included silver nitratre (AgNO_3_ > 99%, Sigma Aldrich) and three Ag NMs, *i.e.*, non-coated Ag-NPs (99%, 20–30 nm, American Elements, further referred as Ag-NPs Non-coated), Polyvinylpyrrolidone (PVP)-coated Ag-NPs (99%, 20–30 nm, American Elements, further referred as Ag-NPs PVP-coated) and Ag NM300K (10.2% w/w Ag, 15 nm; JRC Repository, European Commission, EU). The Ag NM300K is dispersed in 4% polyoxyethylene glycerol triolaete and polyoxyethylene (20) sorbitan mono-laurate (Tween 20), thus the dispersant was also tested alone. The materials’ characteristics are summarized in Table 1.

### 2.4. Spiking Procedure

For Cu materials, spiking was done in soil from control area. All the Cu materials tested (salt and nano) were added to soil as powder, following the OECD recommendations for the testing of non-soluble substances [14]. In short, the materials as dry powders were mixed manually with the dry soil to obtain the corresponding concentration range. After that, deionised water was added to reach 50% of the soil WHC followed by thorough mixing. The procedure was performed for each replicate individually. The soil contaminated in the field—Hygum site (test condition Cu-Field) was collected along the Cu contamination range at the final Cu concentrations of 502 and 1398 mg Cu/kg (measured by Atomic Absorption Spectroscopy).

For the Ag materials, the Ag-NPs PVP-coated and Ag-NPs Non-coated were added to the soil as powder, following the procedure previously described for Cu materials. AgNO_3_, Ag NM300K and the dispersant being soluble or dispersed respectively, were added to the pre moistened soil as aqueous dispersions.

**Table 1:** Summary information of the characteristics of the tested materials in terms of supplier, state, solubility, coating, nominal size (according to the supplier), size according to Transmission or Scanning Electron Microscopy (TEM or SEM) measurements, purity and morphology. PVP: polyvinylpyrrolidone, w/w: wet weight.

Characteristic	COPPER			SILVER				
	CuNO_3_	Cu-NPs	Cu-Nwires	AgNO_3_	Ag-NPs Non-Coated	Ag-NPs PVP-coated	Ag NM300K	Dispersant (Tween 20)
**Supplier**	Sigma Aldrich	American Elements	Synthesized [17]	Sigma Aldrich	AG-M-03M-NP.020N	AG-M-03M-NPCP.020N	JRC Repository	JRC Repository
					(American Elements)	(American Elements)		
**State**	Powder	Powder	Powder	Powder	Powder	Powder	Suspension	Suspension
**Solubility**	Water soluble	Not dispersed	Not dispersed	Water soluble	Not dispersed	Not dispersed	Dispersible	Soluble
**Coating**	-	-	-	-	-	0.2%w/w PVP	-	-
**nominal size (nm)**	-	20–30	90–120 nm diameter, 40–50 µm length	-	20–30	20–30	15	-
**TEM/SEM (nm)**	-	n.a.	365 ± 100 nm diameter, > 10 µm length	-	26 ± 4	25 ± 5	17 ± 8	-
**Purity**	>99%	99.8%	-	>99%	99%	99%	10.2% w/w Ag	-
**Morphology**		flower like *	wires		Spherical *	Spherical *	Spherical	-

* Agglomerates observed.

The tested concentrations were selected based on the known reproduction effect concentrations EC_20_ and EC_50_, for *E. crypticus* within the 95% of confidence intervals (see Table 2).

**Table 2:** Exposure concentrations (values in mg/kg) based on the reproduction effect concentrations EC_20_ and EC_50_ estimates [18,19]. * Tween 20: 4% w/w dispersant.

Material Tested	Control-EC_20_-EC_50_ (mg/kg)
**COPPER**	
CuNO_3_	0-290-360
Cu-NPs	0-980-1760
Cu-Nwires	0-850-1610
Cu-Field	0-500-1400
**SILVER**	
AgNO_3_	0-45-60
Ag-NPs PVP-coated	0-380-550
Ag-NPs Non-coated	0-380-430
Ag NM300K	Dispersant *-60-170

Four biological replicates were performed per test condition, including controls. For Cu exposure, the control group (for all the Cu treatments) consists of soil from control area at Hygum site with a Cu background concentration of 15 mg/kg [16]. For Ag exposure, two control sets were performed: CT (un-spiked LUFA soil, the control condition for AgNO_3_, Ag PVP-Coated and Ag Non-Coated treatments) and CTd (LUFA soil spiked with the dispersant Tween 20, the control condition for the Ag NM300K treatments).

### 2.5. Experimental Details

Testing followed the OECD guideline [13,14] with adaptations as follows. Thirty adult enchytraeids [20] with developed *clitellum* and similar size were introduced in each test vessel containing 20 g of moist soil (50% WHC). The organisms were exposed for 3 and 7 days under controlled conditions of photoperiod (16:8 h light:dark) and temperature (20 ± 1 °C) without food. After the exposure period, the organisms were carefully removed from the soil, rinsed in deionised water and frozen in liquid nitrogen. The weight of each sample (pool of 30 enchytraeids) was recorded and the samples were stored at −80 °C until further analysis. Organisms from day 0 of exposure (collected from cultures) were also sampled. Prior the analysis, each replicate was homogenized in 1000 µL of ultra-pure water and divided in three sub-samples of 300 µL, *i.e.*, (1) for total protein and carbohydrate content determination; (2) for total lipid content determination and (3) for the electron transport system (ETS) activity.

### 2.6. Energy Available—Ea

Available energy reserves were measured by spectrophotometric quantification of the total lipid, protein, and carbohydrate content as described in De Coen and Janssen [9]. The method was applied as described in Novais and Amorim [11]. Total lipids were extracted from 300 µL of homogenized sample according to the method described by Bligh and Dyer [21] and total lipid content was determined by measuring the absorbance at 400 nm using Tripalmitine (Sigma Aldrich, St. Louis, MO, USA) as standard. Total protein and carbohydrate contents were determined according to De Coen and Janssen [9]. Carbohydrate determination was done with phenol 5% and concentrated H_2_SO_4_ at 492 nm using glucose as standard. Protein content was determined according to the Bradford method [22] at 592 nm using bovine serum albumin as standard. The different energy fractions, were converted into energy contents by multiplying the nutrient contents by the respective energetic equivalents using enthalpy combustion (24 kJ/g proteins, 17.5 kJ/g carbohydrates and 39.5 kJ/g lipids) [8,9].

### 2.7. Energy Consumption—Ec

The energy consumption (consumed oxygen rate) was determined based on the measurement of the electron transport system (ETS) activity [23], following the methodology described in detail in De Coen and Janssen [9]. ETS activity was measured in 300 µL of homogenized by adding NADPH solution and INT (*p*-iodonitrotetrazolium, Sigma Aldrich) and following the increase in absorbance at 490 nm for 3 min. The oxygen consumption rate (Ec) was determined based on the theoretical stoichiometrical relationship that for each 2 µmol of formazan formed, 1 µmol of O_2_ was consumed in the ETS system [9]. This amount of consumed oxygen was then transformed into energetic equivalents using the specific oxyenthalpic equivalents for an average lipid, protein and carbohydrate mixture of 480 kJ/mol O_2_ [24].

### 2.8. Cellular Energy Allocation—CEA

The total Ea value was calculated by integrating the change in the summed energy reserves fractions (protein, carbohydrate and lipid budgets) over the two exposure periods (0–3 days and 3–7 days). The Ec value was similarly calculated, integrating its change over the same exposure periods. The CEA, representing the total net energy budget, was calculated for each time interval as described in De Coen and Janssen [9], using the following equation:

(1) $$CEA\;\left(mJ\;{mg}^{-1}\;{Org}^{-1}\right)=\;\frac{\int_{t-1}^{t}Ea.dt-\int_{t-1}^{t}Ec.dt}{T-\left(Tt-1\right)}$$

with *t* being the exposure time (3 or 7 days) and T*t-1* being the previous time in which measurements were done (e.g., if *t* is 7 days, then *t-1* is 3 days).

### 2.9. Statistical Analysis

Significant differences between controls and each treatment (Cu or Ag material), from each exposure period, were determined by one-way analysis of variance (ANOVA), after testing data for normality (Kolmogorov-Smirnov test) and homogeneity variance (Levene’s test). Whenever significant differences were obtained, the post hoc Dunnett’s method for multiple comparisons was used with 95% confidence level (SigmaPlot 11.0, Systat Software Inc., San Jose, CA, USA).

Since the data can also be displayed in a multivariate outline, although only 3 dimension, the relationship between the three parameters of Ea (Proteins, Carbohydrate and Lipid budgets) was investigated using Principle component and Canonical Correspondence analyses, (SAS 9.1.3, SAS Institute Inc., Cary, NC, USA), however no clear patterns were observed and this approach is not further discussed.

## 3. Results

### 3.1. Copper Exposure

**Figure 1:**
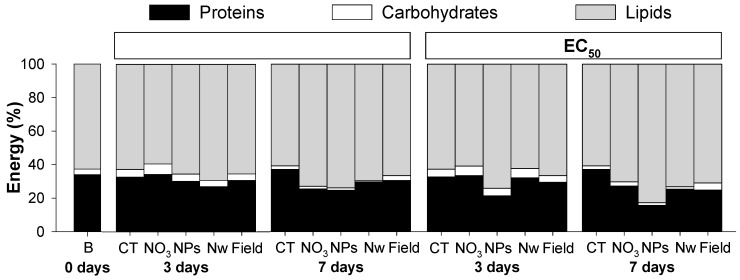
Proportion of energy reserves available (proteins, carbohydrates and lipids) in *Enchytraeus crypticus* after exposure to the concentrations of effect on reproduction EC_20_ and EC_50_ of copper nitrate (NO_3_), copper nanoparticles (NPs), copper nano-wires (Nw) and copper-salt from field contamination (Field), for 0, 3 and 7 days. CT: control, B: Baseline (organisms from cultures).

In control conditions, the weight of the organisms increased during the first 3 days and stabilized from 3 to 7 days (W_0d_ = 0.19 ± 0.01; W_CT3d_ = 0.27 ± 0.05; W_CT7d_ = 0.26 ± 0.05 g/organism). For Cu exposure there were in general no changes in weight, except for a weight decrease in the EC_20_ of Cu-NPs for 7 days (W_Cu-NPs_EC20_7d_ = 0.19 ± 0.02 g/organism). The proportion of each element of Ea (protein, carbohydrates, lipids: P-C-L) at 0, 3 and 7 days of exposure, is shown in Figure 1 (and Appendix Table A1).

Overall, results showed that, in controls, lipids contributed with the highest proportion to the Ea (more than 60%), followed by proteins (20% to 30%) and finally carbohydrates (less than 10%). From 3 to 7 days of exposure to Cu there was a decrease in carbohydrates across all the conditions. The major difference for Cu exposed organisms, in comparison to CT, was a clear general reduction in protein content with increased exposure time and concentration (Figure 1).

**Figure 2:**
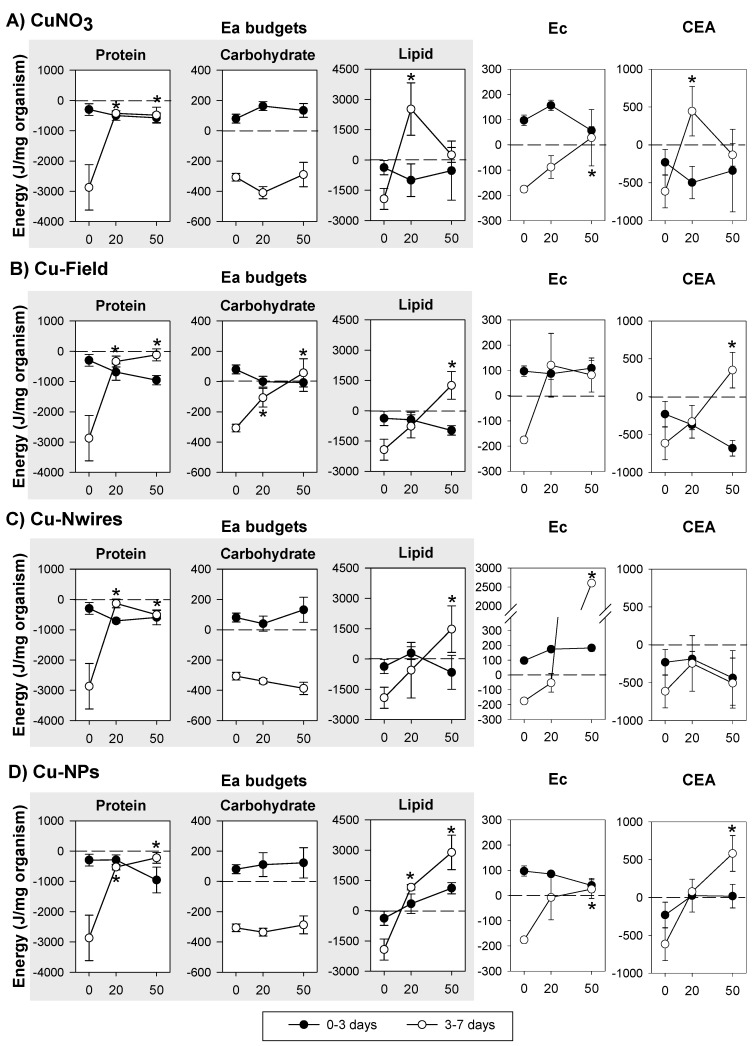
Effects of the reproduction effect concentrations (EC_20_ and EC_50_) of (**A**) copper nitrate—CuNO_3_; (**B**) copper historical contamination—Cu-Field; (**C**) copper nanowires—Cu-Nwires; and (**D**) copper nanoparticles—Cu-NPs, on the Energy available budgets (Ea: in terms of protein, carbohydrate and lipid budgets), Energy consumption (Ec) and Cellular Energy Allocation (CEA) in *Enchytraeus crypticus*, within the exposure periods (0–3 and 3–7 days). Results are expressed as average ± standard error. * Dunnett’s test, *p* < 0.05, for differences between EC_x_ and control within 3–7 days. 0: control, 20: EC_20_, 50:EC_50_.

The integration of the results over time in terms of energy budgets (Ea, Ec and CEA) of organisms exposed to the several Cu forms is shown in Figure 2. Regarding Ea (protein, carbohydrate and lipid budgets), in control conditions, all the parameters measured decrease from 3 to 7 days. For Cu exposure no significant changes were observed from 0–3 days, whereas from 3–7 days there was a dose-dependent increase in protein and lipid budgets across all Cu forms. For carbohydrates this does not occur except for Cu-Field which increased in a dose-dependent manner.

Ec significantly increased from 3 to 7 in all the Cu forms. The Ec for the EC_50_ Cu-Nwires exposed organisms had a particular large change unseen for other materials. CEA tended to increase in all except Cu-Nwires (3–7 days).

### 3.2. Silver Exposure

There were no differences in the weight of the organism between the two control groups (CT and CTd) within each time of exposure. There was a significant decrease in the body weight of the organisms at day 7 in control conditions (W_CT + CTd 7d_ = 0.31 ± 0.08 g/organism) in comparison to organisms from day 0, collected from the cultures (W_0d_ = 0.55 ± 0.07 g/organism). Exposure to Ag caused no changes in weight, compared to controls.

**Figure 3:**
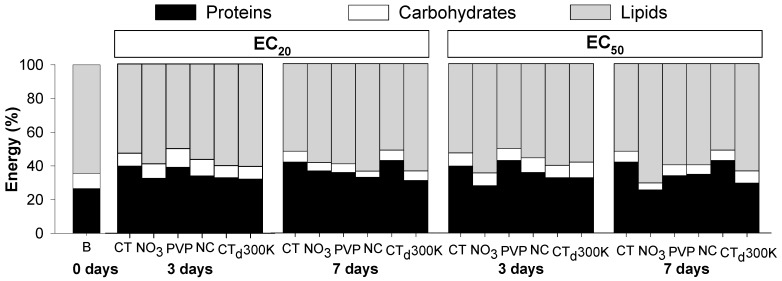
Proportion of energy reserves available (proteins, carbohydrates and lipids) in *Enchytraeus crypticus* after exposure to the concentrations of effect on reproduction EC_20_ and EC_50_ of silver nitrate (NO_3_) and three silver nanoparticles: Ag PVP-Coated (PVP), Ag Non-Coated (NC) and Ag NM300K (300K) for 0, 3 and 7 days. CT: control, CTd: control dispersant, B: Baseline (organisms from cultures).

Figure 3 (and Appendix Table A2) shows the proportion of each element of the Ea at days 0, 3 and 7. Results showed that in control conditions, there is an increase in proteins (from 30% to 40%) with decrease in lipids proportion (from 60% to 50%) in comparison with organisms from the cultures (day 0) (Figure 3). In controls, lipids contributed with more than 50% of the energy reserves available, followed by proteins (around 40%) and carbohydrates with less than 10%. Organisms exposed to Ag from 3 to 7 days had a decrease in the carbohydrate levels (clearest in the EC_20_). The most notorious change was the decrease in protein levels with increase in lipid levels caused by AgNO_3_ (most pronounced at the EC_50_ level) and to some extent also for Ag NM300K (Figure 3)_._

**Figure 4:**
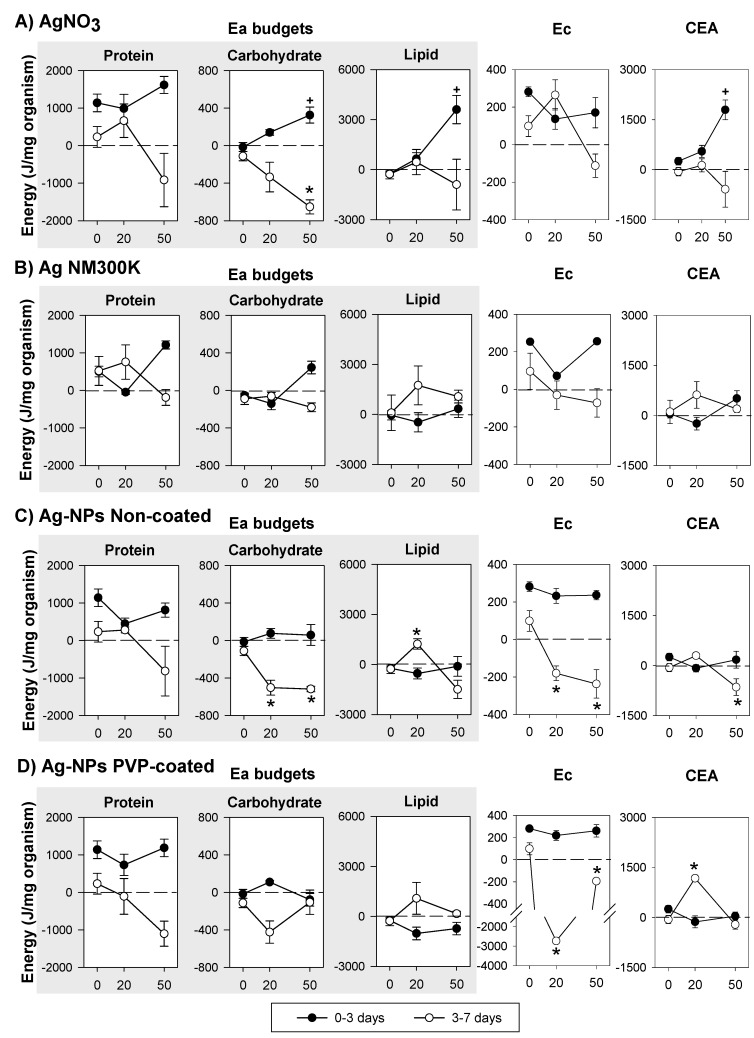
Effects of the reproduction effect concentrations (EC_20_ and EC_50_) of (**A**) silver nitrate—AgNO_3_; (**B**) dispersed silver nanoparticles—Ag NM300K; (**C**) Non-coated silver nanoparticles—Ag-NPs Non-coated; and (**D**) PVP-coated silver nanoparticles—Ag-NPs PVP-coated, on the Energy available budgets (Ea: in terms of protein, carbohydrate and lipid budgets), Energy consumption (Ec) and Cellular Energy Allocation (CEA) in *Enchytraeus crypticus*, over periods of exposure (0–3 and 3–7 days). Results are expressed as average ± standard error. ⁺,* Dunnett’s test, *p* < 0.05, for differences between EC_x_ and control within 0–3 days (⁺) and 3–7 days (*). 0: Control, 20: EC_20_, 50: EC_50_.

The results on the net energy budget (including Ea, Ec and CEA) for Ag exposure can be seen in Figure 4. In controls, Ea budgets tended to decrease with increase in exposure duration, except for Ag NM300K (Figure 4). For all Ag exposures, protein budget showed no significant change from 0 to 3 days of exposure, although there seem to be a decrease at the EC_20_ followed by an increase at the EC_50_ for the Ag-NPs Non-coated. From 3 to 7 days there was a clear decrease in protein budget at the EC_50_ of Ag exposed organisms, in comparison with the control. Carbohydrate budget increased, from 0 to 3 days, after exposure to EC_50_ AgNO_3_ and Ag NM300K. From 3 to 7 days, carbohydrate budget decreased in AgNO_3_ and Ag-NPs non-coated exposures. Lipid budgets increased in AgNO_3_ (0–3 days), whereas from 3 to 7 days, the lipid budget increased at the EC_20_ followed by a decrease at the EC_50_ for the Ag-NPs Non-coated, showing a similar trend for the other Ag forms. Regarding Ec, no changes occurred from 0 to 3 days, but from 3 to 7 days there was a decrease in Ec, except for AgNO_3_ EC_20_. CEA was significantly increased by AgNO_3_ EC_50_ (0–3 days). From 3 to 7 days, CEA was significantly increased by Ag-NPs PVP-coated EC_20_ and significantly decreased by Ag-NPs Non-coated EC_50_.

## 4. Discussion

For both Cu and Ag exposure, the energy content distribution (P-C-L) is in the same range of what was found for other invertebrates (e.g., the ground beetle *Pterostichus oblongopunctatus* [25], the mussel *Mytilus galloprovincialis* [26] and the enchytraeid *E. albidus* [11,27]) with lipids and proteins contributing with the highest proportion of Ea. Nevertheless, variations in the proportions of P-C-L can occur even within the same species (e.g., in *E. albidus* proteins varying from 40% to 60% and lipids from 50% to 40% [10,11]), as observed here.

In control conditions of both Cu and Ag exposures, there was a decrease in all Ea parameters measured over time. This could be related with the fact that the organisms were not fed during the exposure. This is not critical for short exposure periods and allows the quantification of the metabolic use of energy reserves without the interference of effects on feeding behaviour and energy intake [28].

### 4.1. Copper Exposure

Common to all Cu forms is the tendency to cause protein budget decrease from 0 to 3 days followed by its significant increase at 3–7 days (Figure 2) in comparison to the respective control. As proteins are considered constitutive components (while carbohydrates and lipids are storage type) [26] their mobilization is not expected to occur unless under strong stress situations and/or long periods of exposure [29], a mobilization which seem to be the case in the present study. Nevertheless, protein budget depletion was also observed after short term exposure (0–2 days) to EC_10_ and EC_20_ dimethoate in *E. albidus* [11]. On the other hand, the increase in protein budget from 3–7 days of exposure may reflect an induction in protein synthesis for detoxification processes [30], e.g., Cu-salt and Cu-NPs cause the activation of proteins/enzymes involved in the anti-oxidant defence mechanisms [4,31].Whether the same mechanism apply across the materials here (e.g., NMs and salts) is not clear, although it is known that elemental Cu-NPs may oxidise to Cu I oxide and Cu II oxide [32,33] which may lead to a release of Cu ions. Such an oxidation layer also works as a barrier to further oxygen diffusion inhibiting release of ions [18,32]. Hence, some of the similarities across the materials maybe due to released ions, but the effect may also be NPs specific; as also reported in several studies e.g., [4,33,34,35].

The increase in lipids budget (3–7 days) with increasing concentration, common among the Cu forms tested, is similar to the observation by Einicker-Lamas *et al.* [36], who observed a lipid accumulation in *Euglena gracilis* exposed for 72 hours to lethal doses (LD_50_) of CuCl_2_. Similarly, an in vitro study using liver cells [37] also showed an increase in lipid bodies in the cells exposed for 12 h to Cu. Kennedy *et al.* [37] also demonstrate that the rate of lipid accumulation was dependent on the ligand environment of Cu, *i.e.*, Cu complexes of Cu-histidine and Cu-EDTA caused more lipid accumulation than CuCl_2_ (free Cu). In the present experiment the ligand is likely organic matter and clays [18,38]. Despite the similar trends in increased lipid budgets observed in the present study, there was a large difference in terms of absolute values of the lipid budgets; the EC_50_ of Cu-NPs exposed organisms reaching >3000 J/mg organism followed by Cu-Nwires and Cu-Field with 1500 and 1000 J/mg organism, respectively, and CuNO_3_ causing a shift from 3000 to around 0 J/mg organism from the EC_20_ to the EC_50_. Since the exposures are based on similar EC_x_ values across materials, it is not likely that these trends are due to possible availability of free ions (although time may be an issue).

Carbohydrate budget was comparatively reduced from 0–3 days to 3–7 days by CuNO_3_, Cu-NPs or Cu-Nwires exposure, while Cu-Field caused an increase in a dose-dependent way (3–7 days). Similar pattern, *i.e.*, increase or no alterations in carbohydrate budget in comparison to control, was also observed in other organisms responses’ to sub-lethal concentrations of metals (e.g., in *Daphnia magna* exposed to Hg [9] or Cd [8], for concentrations higher than the reproduction EC_50_ a reduction is reported. In regard to the Cu-Field something clearly different occurred, a dose-dependent increase in carbohydrate budget (3–7 days). Hyperglycemia (increase in “blood” glucose levels) is a common response to metals and other stressor in aquatic crustaceans [39] and was also found in the earthworm *Aporrectodea longa* in response to oil based drilling mud (lubricant used in oil drilling operations) in soil [40]. It was suggested [40] that the increase in glucose levels could be due to intensive glycogenolysis (production of glucose using glycogen), but in the present work we measured total carbohydrates and a reduction in glycogen would be reflected in the total carbohydrate budget. On the other hand, a scenario of gluconeogenesis (production of glucose from non-carbohydrate sources) might be occurring. Why this is only the case for the Cu-Field soil is unknown.

The increase in metabolic activity (Ec increased in all the Cu forms tested (3–7 days)) reflects the increased energetic demand to deal with the stress caused by Cu. This has been observed for other organisms exposed to metals (e.g., *E. albidus* to Cd and Zn [10], *Pterostichus oblongopunctatus* to Ni [41]) and the increase in metabolic rate is a common effect of Cu exposure in aquatic organisms (clamps, fish and crabs) [42,43,44]. Muller *et al.* [45] found that ZnO NPs cause an increase in mussels’ respiration rate and conclude that the NPs target the maintenance of the organisms (instead of growth). In our study, this increase in Ec may also be related with the increase in the protein synthesis which is an energy demanding process. As observed for lipid budgets, the Ec increase is not similar across Cu materials, with much higher values being reached for Cu-Nwires (3000 *vs.* 300 J/mg). These results indicate that organisms have different energy requirements to deal with the stress caused by the different Cu forms. One clear difference is that Cu-Nwires oxidize much more than Cu-NPs, which must induce the activation of distinct mechanisms of detoxification maybe related to the high aspect-ratio of the Nwires. The difference in response between the Cu-NPs and the Nwires is maybe particularly striking since not only are they compared on similar effect levels (EC_x_ values), but the external concentration (total Cu in soil) were also almost identical (see Table 2).

The decrease in the net energy budget of an organism (CEA) can result from a reduction in energy reserves available and/or the increase in energy consumption [8,12]. Here, Ec increased from 3 to 7 days of exposure to the several Cu forms, but none of the parameters of Ea were significantly reduced. On the contrary, the observed increase in protein and lipids probably compensated the increase in Ec, thus resulting in the increased CEA observed. Other authors found increased CEA values [9,11]. Our results suggests that for Cu (in agreement with Novais and Amorim [11]), reproduction EC_20_ and EC_50_ (3 weeks) could not be directly linked to a CEA reduction (7 days).

### 4.2. Silver Exposure

The Ea pattern of an increased protein budget (in comparison to control) at lower doses followed by its decrease at higher doses (as observed from 3 to 7 days) in response to AgNO_3_ and Ag NM300K (and for the Ag-NPs PVP-coated and Ag-NPs Non-coated without the clear increase at the EC_20_) has also been reported for several other stressors in *D. magna* [8]. According to Smolders *et al.* [12] low levels of stress can trigger increase in protein synthesis for detoxification processes if carbohydrates and lipids are available as energy sources. In fact, at similar effect concentrations and period of exposure (*i.e.*, exposure to the EC_20_ from 3–7 days) there was a depletion of carbohydrate budgets in AgNO_3_, Ag-NPs PVP-coated and Ag-NPs Non-coated treated organisms while the lipid budget increased, indicating that carbohydrates were being used as the primary energy source. The mobilization of carbohydrate reserves in response to metal exposure is well documented (e.g., [8,10,46,47,48,49]) and has often been referred as the first and rapidly available energy source used under stress conditions [30,50,51]. The use of carbohydrates was not observed for Ag NM300K, in fact, protein budget was the only Ea which decreased during Ag NM300K exposure (Figure 4B). As mentioned before, the decrease in protein reserves observed for all the Ag forms is not unusual, and according to Boeck *et al.* [46] might result from the induction of gluconeogenesis to keep glucose levels, thus stimulating protein catabolism. Interestingly, for those treatments where the carbohydrate budget decreased (AgNO_3_, Ag-NPs PVP-coated and Ag-NPs Non-coated), the protein budget depletion was in general higher than for Ag NM300K, probably because proteins are being mobilized to compensate the reduction in carbohydrates.

Lipid budget was increased in response to AgNO_3_ EC_50_ (0–3 days) and to Ag-NMs (Ag NM300K, Ag-NPs Non-Coated and Ag-NPs PVP-coated) EC_20_ (3–7 days), which fits with that lipid accumulation has been associated with inflammatory stress (e.g., [52]), a response type also observed for AgNO_3_ and Ag-NPs exposures [53]. The fact that lipids accumulated earlier for AgNO_3_ than for Ag-NMs exposure could indicate that either (i) a slower uptake of Ag-NMs but otherwise a similar effect between the two [54]; or (ii) that the effects in the NMs exposed organisms were caused by slower Ag ions release in the soil (e.g., [55,56]) or intracellular [57]. Longer exposure period (3–7 days) for the AgNO_3_ EC_50_ caused a decrease in lipid budget, while for the Ag-NMs at the EC_50_ the lipid budget returned to control levels (Ag-NPs PVP-coated), decreased (Ag-NPs Non-coated), or remain elevated (Ag NM300K). At least two possibilities arise from these results (or the combination of both) *i.e.*, the NMs are internalized differently in the cells, or the NMs have different dissolution rates causing enchytraeids to be exposed differently to Ag ions over time. However, if ions are released over time in the soil, then the sorption to the organic matter or clay would likely make them unavailable to the organisms.

The decrease (for all Ag forms) in Ec might be linked with mitochondrial damage with subsequent disruption of its respiratory chain [58,59,60], that has been observed for AgNO_3_ and Ag-NPs (e.g., [61]). With exception of Ag PVP-coated EC_20_ (where Ec was much more reduced than any other treatment or the EC_50_) all other caused a similar reduction on Ec, so unlike observations for lipid budgets where a temporal delay between Ag-salt and NMs seemed to occur, for Ec the effects might relate to the population responses (*i.e.*, impairment of reproduction). The difference in response between the Ag-NPs PVP-coated and Ag-NPs Non-coated is maybe particularly striking since not only are they compared on similar effect levels (EC_x_ values), but the external concentration (total Ag in soil) were also almost identical (see Table 2).

### 4.3. Copper vs. Silver

For both Cu and Ag (at EC_20_) a decrease in carbohydrate levels at 7 days of exposure was observed; this was also observed for the EC_50_ of Cu materials but not for the EC_50_ of Ag materials. Interestingly, for some of the Ag materials there was a decrease in carbohydrate budgets, whereas for Cu materials the decrease in carbohydrate proportion was not reflected in the carbohydrate budgets. Clear opposite trends in the other energy reserves were observed with the protein budget increasing for the Cu materials and decreasing for Ag materials, while lipid budget tended to increase in both. The results, as discussed, could be linked with known mechanisms of toxicity for Cu and Ag; for instance, the increased metabolic rate in Cu exposed animals [42,43,44] and the impairment of mitochondria, with disruption of its respiratory chain in response to Ag [58,59,60]. Overall, the CEA parameters showed a clear distinction between Cu and Ag materials exposure.

## 5. Conclusions

Exposure to the various Cu and Ag materials affected the energy metabolism of *E. crypticus*. Overall, these effects were discriminated between Cu and Ag materials indicating the differences in mechanisms. This is not surprising given the differences as essential versus non-essential. Moreover, there was a clear identification between effects of Cu and Ag, in line with known mechanisms of Cu and Ag toxicity. The increase in energy demand to respond to Cu stress could be associated with the increase in protein synthesis required for detoxification. For Ag, mitochondrial damage is reflected in the reduction of Ec and can explain damage to other biomolecules (e.g., proteins) as caused by ROS formation. Lastly, differences were observed between the various nano and non-nano forms tested which clearly highlights the differences in mechanisms. For Ag, results are mostly in line with the slower oxidation rate effect of Ag-NMs compared to Ag-salt (ions). For Cu, the various materials seem to trigger different mechanisms of response and size and shape have a clear role in the effects.

## Appendix

**Table A1:** Proportion (%) of energy reserves available (proteins, carbohydrates and lipids) in *Enchytraeus crypticus* after exposure to the concentrations of effect on reproduction EC_20_ and EC_50_ of copper nitrate (CuNO_3_), copper nanoparticles (Cu-NPs), copper nano-wires (Cu-Nwires) and copper-salt from field contamination (Cu-Field), for 0 days (Baseline), 3 and 7 days. CT: control.

Time	EC_x_	Treatment	% Proteins	% Carbohydrates	% Lipids
0 days		Baseline	34.1	3.2	62.7
3 days	EC_20_	CT	32.8	4.6	62.7
		CuNO_3_	34.3	6.2	59.4
		Cu-NPs	30.2	4.3	65.5
		Cu-Nwires	26.9	3.8	69.2
		Cu-Field	30.7	3.9	65.4
	EC_50_	CT	32.8	4.6	62.7
		CuNO_3_	33.5	5.6	60.9
		Cu-NPs	21.5	4.4	74.1
		Cu-Nwires	32.2	5.5	62.3
		Cu-Field	29.5	4.0	66.5
7 days	EC_20_	CT	37.2	2.1	60.7
		CuNO_3_	25.6	1.5	72.9
		Cu-NPs	24.7	1.5	73.8
		Cu-Nwires	29.8	0.8	69.5
		Cu-Field	30.6	2.8	66.6
	EC_50_	CT	37.2	2.1	60.7
		CuNO_3_	27.2	2.5	70.3
		Cu-NPs	15.7	1.6	82.7
		Cu-Nwires	25.4	1.4	73.2
		Cu-Field	24.9	4.3	70.9

**Table A2:** Proportion (%) of energy reserves available (proteins, carbohydrates and lipids) in *Enchytraeus crypticus* after exposure to the concentrations of effect on reproduction EC_20_ and EC_50_ of silver nitrate (AgNO_3_) and three silver nanoparticles: Ag PVP-Coated, Ag Non-Coated and Ag NM300K for 0 days (Baseline), 3 and 7 days. CT: control.

Time	EC_x_	Treatment	% Proteins	% Carbohydrates	% Lipids
0 days		Baseline	26.6	8.7	64.7
3 days	EC_20_	CT	39.5	7.8	52.7
		AgNO_3_	32.4	8.6	59.0
		Ag PVP-Coated	38.9	11.1	50.0
		Ag Non-Coated	33.7	9.9	56.4
		CT dispersant	32.7	7.2	60.1
		Ag NM300K	31.9	7.6	60.5
	EC_50_	CT	39.5	7.8	52.7
		AgNO_3_	28.0	7.5	64.5
		Ag PVP-Coated	42.9	7.0	50.1
		Ag Non-Coated	35.7	8.6	55.7
		CT dispersant	32.7	7.2	60.1
		Ag NM300K	32.7	9.1	58.2
7 days	EC_20_	CT	41.9	6.4	51.7
		AgNO_3_	36.6	5.0	58.4
		Ag PVP-Coated	35.7	5.1	59.2
		Ag Non-Coated	32.9	3.6	63.5
		CT dispersant	42.8	6.2	51.0
		Ag NM300K	31.0	5.7	63.3
	EC_50_	CT	41.9	6.4	51.7
		AgNO_3_	25.4	4.1	70.4
		Ag PVP-Coated	33.9	6.4	59.7
		Ag Non-Coated	34.7	5.5	59.8
		CT dispersant	42.8	6.2	51.0
		Ag NM300K	29.4	7.2	63.3
